# Chance or Necessity—The Fungi Co−Occurring with *Formica polyctena* Ants

**DOI:** 10.3390/insects12030204

**Published:** 2021-02-28

**Authors:** Igor Siedlecki, Michał Gorczak, Alicja Okrasińska, Marta Wrzosek

**Affiliations:** 1 Mycological Laboratory, Biological and Chemical Research Centre, University of Warsaw, ul. Żwirki i Wigury 101, 02-089 Warsaw, Poland; gorczak@uw.edu.pl (M.G.); alis.ok@biol.uw.edu.pl (A.O.); ma.wrzosek@uw.edu.pl (M.W.); 2 Botanic Garden, Faculty of Biology, University of Warsaw, Al. Ujazdowskie 4, 00-478 Warsaw, Poland; 3 Institute of Evolutionary Biology, Faculty of Biology, University of Warsaw, ul. Żwirki i Wigury 101, 02-089 Warsaw, Poland

**Keywords:** red wood ants, *Penicillium*, *Entomortierella*, mycobiota, ant–fungal interactions

## Abstract

**Simple Summary:**

There are about 13,800 species of ants living around the world, but only some of them have been extensively studied in the context of their non−antagonistic relationships with fungi. The best−known example is the symbiosis between leaf−cutting ants and fungi serving them as food. Others include the relationship between ants living in carton nests in the trees’ canopy with fungi increasing the durability of the nest. Do ants utilize fungi in the northern hemisphere and cooler climatic zone? This question is still open. Our goal was to study the less−obvious interactions between ants and common fungi in temperate climates. In our study, we characterized the mycobiota of the surroundings of *Formica polyctena* ants. We identified nearly 600 strains and investigated their taxonomic affinity. The most abundant fungi in *F. polyctena* nests are strains belonging to *Penicillium*—a genus well−known as an antibiotic producer. Other common and widespread fungi related to *Penicillium*, such as the toxin−producing *Aspergillus* species, were isolated very rarely. Additionally, the high diversity and high frequency of *Penicillium* colonies isolated from ants in this study suggest that certain representatives of this genus may be adapted to survive in ant nests, or that they are preferentially sustained by the insects.

**Abstract:**

Studies on carton nesting ants and domatia−dwelling ants have shown that ant–fungi interactions may be much more common and widespread than previously thought. Until now, studies focused predominantly on parasitic and mutualistic fungi–ant interactions occurring mostly in the tropics, neglecting less−obvious interactions involving the fungi common in ants’ surroundings in temperate climates. In our study, we characterized the mycobiota of the surroundings of *Formica polyctena* ants by identifying nearly 600 fungal colonies that were isolated externally from the bodies of *F. polyctena* workers. The ants were collected from mounds found in northern and central Poland. Isolated fungi were assigned to 20 genera via molecular identification (ITS rDNA barcoding). Among these, *Penicillium* strains were the most frequent, belonging to eight different taxonomic sections. Other common and widespread members of Eurotiales, such as *Aspergillus* spp., were isolated very rarely. In our study, we managed to characterize the genera of fungi commonly present on *F. polyctena* workers. Our results suggest that *Penicillium*, *Trichoderma*, *Mucor*, *Schwanniomyces* and *Entomortierella* are commonly present in *F. polyctena* surroundings. Additionally, the high diversity and high frequency of *Penicillium* colonies isolated from ants in this study suggest that representatives of this genus may be adapted to survive in ant nests environment better than the other fungal groups, or that they are preferentially sustained by the insects in nests.

## 1. Introduction

### 1.1. Mutualistic Fungus–Ant Interaction in the Holarctic Region Is Understudied

Although nonpathogenic interactions between ants and fungi have been studied for many years, most of the previous studies focused on Attine ant–fungal mutualism [1,2,3,4,5]. Contemporary research has revealed some newly described examples of ant–fungal mutualism between “black yeasts” (Chaetothyriales) and arboreal ants that live in domatia on myrmecophytic plants or ants which produce cardboard−like construction material [6,7,8,9,10]. In case of domatia−dwelling ants, the fungal patch cultivated in the nest is a nitrogen−rich food source for ants; “black yeasts” inhabiting the carton walls of nests are not consumed by ants, but they improve the durability of the nest’s structure [6,7,8,10,11]. Until now, most studies on mutualistic relationships between ants and fungi have focused on tropical or subtropical species [3].

In the temperate zone, although the fungal pathogens of ants have been studied extensively [3,12,13,14,15,16,17,18], a non−antagonistic side of ant–fungal interactions remains understudied [3]. Studies searching for such relationships have focused mostly on fungi that inhabit ants’ infrabuccal pockets [19], especially those of *Camponotus* ants [20,21]. Infrabuccal pockets of *Camponotus* ants proved to be commonly occupied by *Schwanniomyces polymorphus* yeasts [21]; additionally, infrabuccal pellets were the substratum from which new species of *Penicillium* and *Mortierella* s.l. were isolated [20,22]. Still, fungal species coexisting in nests and the surrounding environment of Holarctic ant species were investigated sparsely [23,24]. Lindström et al. focused on *F. exsecta* nests’ microbiome and showed that both composition and richness of the microbiome are unique in comparison to the reference soils [24]. For many other ant species, including those that play key roles in the forest ecosystems in temperate climates, the composition of nest−dwelling fungi communities remains unknown.

Ants have developed a lot of diverse adaptations which can negatively impact fungi in their habitats [25,26,27,28,29]. These adaptations can work on an individual level (e.g., prophylactic antifungal compound production by ants’ body glands) or on colony level, through maintaining specific conditions inside the nest [25,26,27,28,30,31]. Nonetheless, given that fungi are highly versatile, it could be assumed that such a specifically shaped environment, while being generally unfavorable for most fungi, can be suitable for certain fungal taxa. Those currently under−investigated fungal species do not necessarily have to act as specialized entomopathogens, but can also affect ants’ biology indirectly and facultatively, acting as commensals or mutualistic partners of ants [32]. Additionally, as was pointed out by Currie, microbes may be key components in the regulation of other symbiotic associations between ants and other organisms [33].

### 1.2. Red Wood Ants’ Mounds Are Very Specific Microenvironments, Actively Shaped by Their Biocenosis

In temperate climates, certain ant species, such as the red wood ants (*Formica* s. str.), have a profound influence on their environment, and thus are often referred to as “ecosystem engineers” [34,35,36]. This group of closely related ant species includes, among others, *F. polyctena*, *F. rufa* and *F. pratensis*, with similar morphology, ecology and biology [34,37]. This ecologically and economically important assemblage inhabits the temperate zones’ mixed and coniferous forests [34,38,39]. European red wood ants create vast colonies (up to millions of individuals), building large, perennial nests out of organic matter [34,38]. Usually, red wood ants’ nests stay active for several dozen years, sometimes even for more than a century [38,39].

A red wood ants’ nest consists of underground and aboveground parts. The former is usually located around a decaying stump, while the latter is a hill made of soil, small stones, and plant−derived organic matter [40]. The organic material incorporated into the nests comes from the adjacent environment and is composed mainly of coniferous trees’ needles [35,36]. Additional building material can be gathered from little twigs, small seeds, and scales from conifer cones. Nests are usually built in sunny spots and oriented in a way that maximizes the exposure to sunlight [41]. Even though red wood ants’ mounds are built mostly with material from their immediate surroundings, the conditions inside them greatly differ from the nearby litter or soil [35].

Several authors have pointed out the outstanding features of mounds, contrasting with the surrounding environments [35,36,38,42,43,44,45]. They include, among others: the accumulation of organic matter (and thus local increase in the availability of nutrients), a shift toward a neutral value of nest pH, increased temperature inside the nest (about 10 °C higher than the ambient temperature), and increased aeration [35,36,38,42,43,44,45]. Importantly, red wood ants’ mounds show high concentrations of resin, which is willingly and continually collected by ants from coniferous trees [26,30,46]. Some authors suggest that the resin is collected by ants for its antibiotic and antifungal properties to decrease microorganisms’ activity or to protect ants from microbial pathogens [26,30,46]. Moreover, Brütsch and colleagues stated that the active spraying of resin with formic acid, performed by red wood ants, further increases the antiseptic properties of the mixture [26].

Because of these differences, red wood ants’ nest is a distinctly separate microenvironment, which may promote formation of a specific microbial community, different than those common for the surrounding forest litter or soil [47,48]. Such a phenomenon was observed for *F. exsecta* [24] (subgenus Coptoformica [34]). The study showed that *F. exsecta* mounds’ core microbiomes differ from the microbiome of the surrounding soils, with *Exophiala*, *Oidiodendron*, *Scleroconidioma*, and *Umbelopsis* being core fungal indicators of the nest [24]. All other studies analyzing the mycobiota of red wood ants’ nests focused only on yeast species; in those studies, yeasts from the Debaryomycetaceae family were commonly isolated from *F. rufa* and *F. aquilonia* nests and were present in nests in much higher quantities than in the surrounding soil [48,49]. Generally, probably due to an increased amount of organic matter in the mound and other features mentioned above, higher quantities of microorganisms are noted for red wood ants’ nests than for the surrounding soil [47,48,50]. The microbial biomass is mostly concentrated in warmer and more moist parts of the hill—the upper part, and in the larval and egg chambers [48,51]. The higher quantity of microorganisms in nests could, however, either be the cause or the effect of such differences.

### 1.3. The Insight into F. polyctena Mycobiota Could Shed a Light on the Complex Interactions between Individuals within and between Nests

One of the most common red wood ant species in European forests is *Formica polyctena*. It is a highly polygynous and polydomous species, with the number of queens reaching thousands, and the number of workers often exceeding one million individuals in one nest [34,40]. Therefore, their mounds are usually big and long−lasting, which offers stable conditions for specific mycobiota to occur. Similarly to other insects [52,53,54], ants collect fungal spores on their bodies while moving and foraging [55,56], and even though they perform grooming behaviors, some fungal spores may remain on their exoskeleton [57,58] or be transported to infrabuccal pockets in ants’ heads [19,25,29,59]. Consequently, fungal spores observed on their bodies’ surface can be regarded as a representative sample of fungi commonly present in the environments of *F. polyctena*.

Despite the remarkable importance of *Formica polyctena* ants in coniferous, temperate forest ecosystems, and their strong local influence on the surrounding environment, there are no studies investigating the mycobiota of this species or their mounds. Therefore, the goal of this study was to identify fungal taxa co−occurring with red wood ants, and to find the taxa which could be described as common cohabitants of ants. We believe that this is the first major step on the way to trace the symbiotic relationships between ants and fungi in temperate climate regions.

## 2. Materials and Methods

In October 2016, red wood ants were collected from 22 mounds located in mixed and coniferous forests of northern and central Poland. Insect specimens (9–30 individuals) were collected from the surface of every mound and placed in sterile containers. They were then identified to the species level using a dissecting microscope (Nikon SMZ800), with the help of identification keys [34,40]. In this study, 328 *Formica polyctena* workers from 18 mounds were analyzed (Table S1). Because all red wood ants are protected in Poland [60], in order to decrease the ecological impact of the study on the colonies, ants were collected without destruction of their nest. The sampling was approved by the Regional Directorate for Environmental Protection (WZG.73.67.2018.APO.1).

In the laboratory, ants were kept until death in sterile, glass containers, in 4 °C. Dead individuals (up to 7 days after death) were placed in Petri dishes on 4% Sabouraud Dextrose Agar (SDA) medium (Fluka, USA). Petri dishes were incubated for 7 days, in 20 °C, in darkness. Grown fungal colonies were identified to a genus level and then assigned to separate morphotypes based on their microscopic and macroscopic features. Fungal colonies representing separate morphotypes were transferred onto fresh media, in order to obtain axenic cultures. The colonies were then identified by the analysis of nucleotide sequences of the ITS rDNA region. Total nucleic acids were extracted using an ExtractMe genomic DNA kit (DNA Gdańsk, Poland), following the manufacturer’s instructions. Amplification of the ITS rDNA regions was carried out using the primers ITS1f [61] and ITS4 [62]. Sequencing was outsourced to an external company, Genomed S.A. (Warsaw, Poland), and the amplified regions were sequenced with the Sanger method using ITS1f primer. In order to identify the isolated strains, obtained sequences were compared with the sequences available in the NCBI GenBank (ncbi.nlm.nih.gov (accessed on 4 January 2021)) database using the BLASTn algorithm [63]. The strains were assigned to known species when their ITS rDNA sequences matched the following conditions: (i) ≥97.0% identity with the matching sequence; (ii) ≥2.0% divergence from the next closest species; and (iii) a matching sequence was submitted by the culture collection or published in a peer−reviewed journal article. If a sequence did not meet all the conditions listed on a given taxonomic level, its identification was concluded on a higher taxonomic level.

The sequences obtained in this study were deposited in the NCBI GenBank database. Voucher specimens were deposited in the Herbarium of the Faculty of Biology, University of Warsaw. GenBank accession numbers and Herbarium collection numbers are available in the Table S2. Four strains for which the ITS sequence could not be obtained were identified solely based on their morphology.

Statistical analyses of obtained data were performed in R v3.6.1 in RStudio using the vegan and phyloseq packages [64,65,66,67]. Visualization methods included built−in functions of Microsoft Excel 365, as well as the ggplot2 R package [68].

## 3. Results

**Figure 1:**
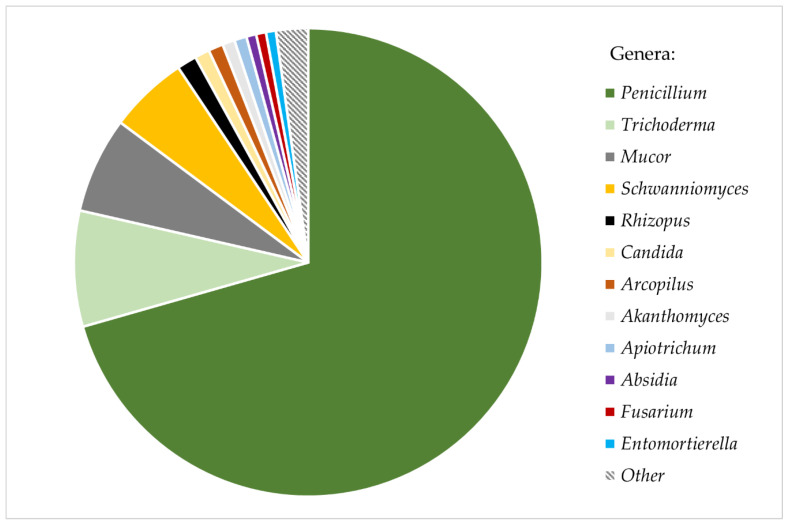
Relative abundance of fungi isolated from bodies of *Formica polyctena* ants.

Nearly all studied ant cadavers (98.48%) were carriers of viable fungal diaspores (spores, mycelium, chlamydospores). A total of 638 fungal colonies were obtained from 323 out of 328 studied ants, yielding an average of 1.98 colonies (σ = 0.39) grown from one ant cadaver. Most colonies (92.16%) were successfully isolated into axenic strains, while the remaining 7.84% of colonies were overgrown by other, faster−growing strains and remained unidentified. In total, fungi belonging to 20 different genera from five subphyla were isolated (Table S2). The majority of the isolated fungi (89.6%) were representatives of the phylum Ascomycota. Colonies identified as *Penicillium* spp. constituted more than 70% of all the isolates. The other most common genera were *Trichoderma* (7.99%), *Mucor* (6.63%), and *Schwanniomyces* (5.44%). All other fungal genera were isolated substantially more rarely from the ant cuticle, each constituting <1.5% of all isolates (Figure 1). Additionally, all *Mortierella* s.l. strains identified represent the newly distinguished genus *Entomortierella* [69]. Some common soil fungi, such as genera *Aspergillus* and *Cladosporium*, were not isolated from ants at all.

**Figure 2:**
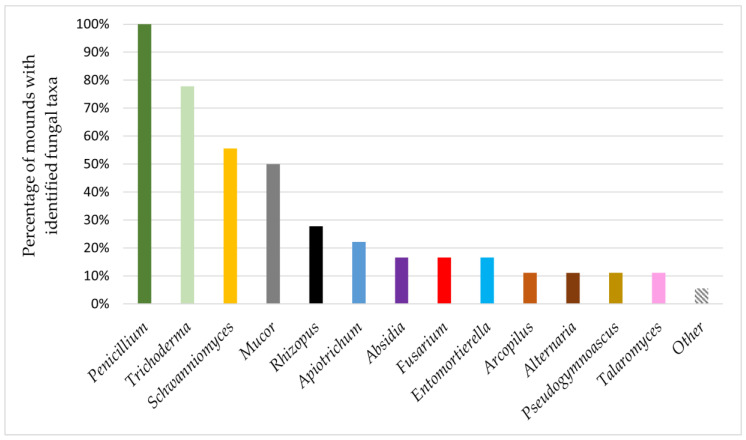
The presence of fungal taxa in *F. polyctena* nests.

Only the representatives of the genus *Penicillium* were present on ants from all studied mounds. Colonies identified as the genus *Trichoderma* appeared on ants from 77.77% of the studied mounds, and representatives of *Schwanniomyces* and *Mucor* were present on ants from 55.55% and 50% of mounds, respectively. Other isolated fungal genera occurred on ants from no more than 27.77% of studied mounds (Figure 2). Representatives of enthomopathogenic fungi were isolated only from one nest and were identified as *Akanthomyces* spp.

**Figure 3:**
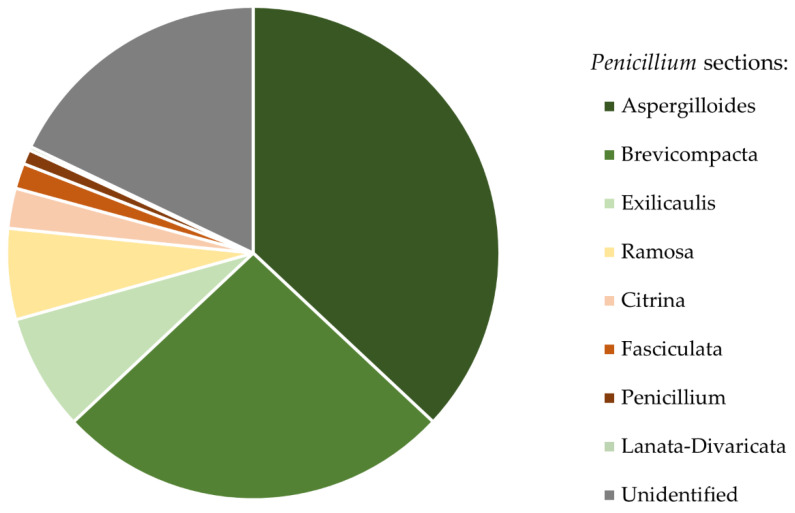
Relative abundance of *Penicillium* strains belonging to different sections, isolated from bodies of *F. polyctena* ants.

ITS rDNA fragments do not allow the identification of *Penicillium* and *Trichoderma* to species level. However, in cases of these genera they do allow for section level identification [70,71]. Thus, we conclude that isolated *Penicillium* colonies belonged to eight sections. Colonies of *Penicillium* sct. Aspergilloides (36.99%) and *Penicillium* sct. Brevicompacta (26.01%) were the most common and occurred on ants from 94.44% of studied mounds (Figure 3). All isolated *Trichoderma* strains belonged to one section: *Trichoderma* sct. Trichoderma. The list of all isolated taxa can be found in Table S2.

**Figure 4:**
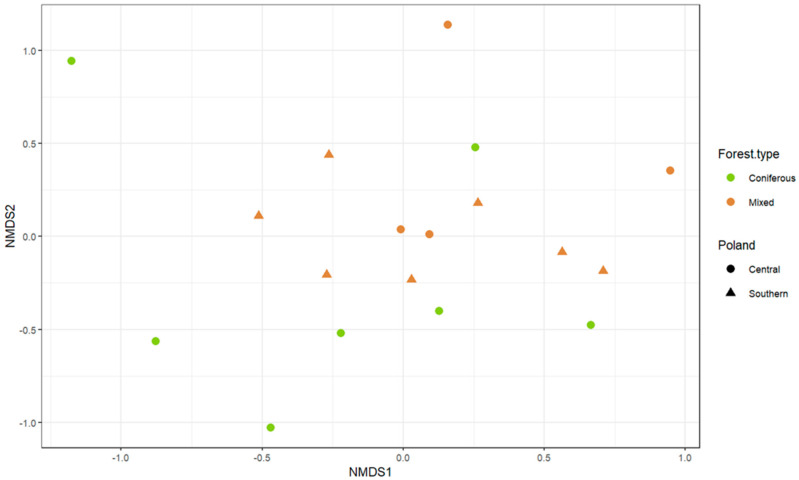
Non−metric multidimensional scaling (NMDS) ordination plot showing fungal communities (numbers of morphotypes within each genus); 2D stress = 0.15. Colors indicate forest type and shapes indicate geographical localization in which the sampled mounds were located.

Differences in taxonomic composition of fungal communities between groups of mounds were assessed using one−way ANOVA on a Bray–Curtis dissimilarity matrix. Mounds were divided into groups based on the type of the surrounding forest (mixed or coniferous) and their geographical localization (northern or central Poland). We observed that the mounds’ fungal community composition differed significantly between forest types (F(1,16) = 2.28; *p* = 0.03), but not between regions (F(1,16) = 0.87; *p* = 0.52); however, none of the groups were visibly well−distinguished on the nMDS ordination plot (Figure 4).

## 4. Discussion

Despite the observation that fungal colonies, most often belonging to the genus *Penicillium*, grew out of almost all the studied *F. polyctena* workers, ants seemed to be relatively clean. Very rarely more than two fungal colonies were isolated from one ant individual. Our observations on the overall scarcity of fungal spores on ants’ bodies suggest that grooming behavior and antifungal substances produced by ants’ glands are rather effective in destruction of the spores. The possibility that *F. polyctena* ants simply do not encounter fungal diaspores in their surroundings would be very unlikely due to the previous observations of increased microbial activity in ants’ nests [26,72] and the high concentrations of fungal spores in the temperate forest environment [73]. Moreover, multiple studies have proven that the grooming behavior is efficient in protection against entomopathogenic fungi by reducing the numbers of spores on the ant integument [27,28]. Well−developed social immunity strategies in ants [27,28] could also be the reason for very low numbers of entomopathogenic fungi isolated in our study, because the only entomopathogenic fungus we found on *F. polyctena* ants belongs to *Akanthomyces*. It is noteworthy that there is only one recorded case of ants’ infection by *Akanthomyces* (on *Solenopis invicta*) [74], while this genus is more broadly known as a lepidopteran [75] and aranean [76] pathogen. Its presence could therefore be indicative of ant predation on infected prey, rather than an actual sign of parasitism. Meanwhile, a different mild entomopathogen—*Aegeritella*, more prevalent on red wood ants [77,78]—could have been present on the studied individuals, but missed in our results due to its slow−growing properties [16].

**Figure 5:**
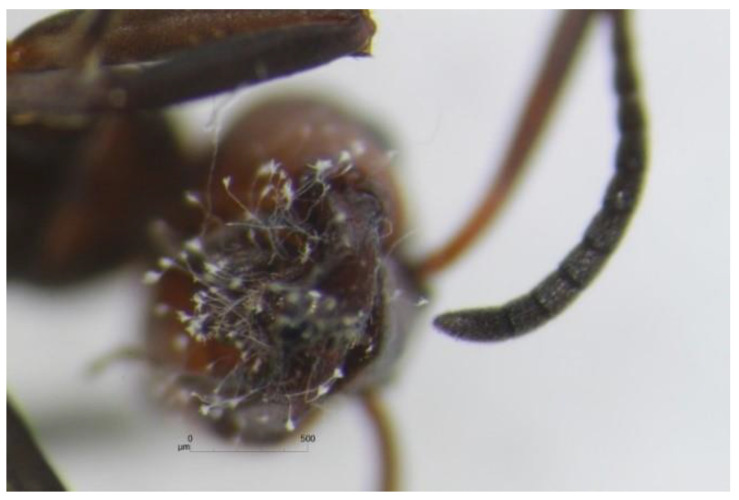
Conidiophores of *Penicillium* growing from the head of a dead *F. polyctena* ant.

In our study, we often observed the fungal colonies to grow from the heads of *F. polyctena* ants (Figure 5). This suggests that the majority of active fungal diaspores are most likely located in the ants’ infrabuccal pockets. This observation is in line with a number of previous studies (e.g., research by Wheeler and Bailey), where authors discovered that fungal structures, in some cases including spores, were commonly present in the infrabuccal pellets of many ant species [19,20,21].

ANOVA results suggest that the forest type is a better predictor of the presence of certain fungal communities in the mounds than those mounds’ geographical localization. The difference between the *F. polyctena* mounds from coniferous and mixed forests may be explained by the variance of organic materials that are collected to build the nests. In mixed forests, more materials from deciduous trees are incorporated into mounds, while in coniferous forests the main construction materials are pine or spruce needles [39]. Moreover, while microscopic fungi have usually broad geographical ranges [79], different fungal taxa are associated with different plant species, their communities, or decaying organic matter originating from them [80,81]. Therefore, it is not unexpected that the ANOVA results suggest that nest mycobiota partially reflects the mycobiota of their respective surroundings. However, grouping based on the forest type is not clearly visible in the NMDS visualization. The lack of strong division between mounds from different forest types may suggest that some fungal diversity is common for mounds from both environments, which could strengthen the hypothesis that the red wood ants’ mound is a specific microenvironment.

Results of this study showed that *Penicillium* and *Trichoderma* representatives were isolated from ants from most of the studied nests. *Penicillium* is a very common and widespread genus [82], with many saprotrophic species able to degrade plant tissue [83]. Therefore, it should be expected that the representatives of this genus were found on red wood ants, which inhabit nests built mostly from plant material. *F. polyctena* nest material is enriched by resin and is regularly sprayed with formic acid [26]; therefore, *Penicillium* species present in red wood ants’ mounds clearly seem to be resistant to these antifungal substances. Interestingly, *Penicillium* representatives have been found to be present in the nests of *F. exsecta* significantly more often than in the surrounding soil [24]. Moreover, there is a growing amount of evidence that *Penicillium* species can co−occur with insects, possibly as facultative symbionts [84,85] and as mutualists [86]. At the same time, there are currently no data indicating that *Penicillium* could be antagonistic toward insects. The results of our studies suggest that representatives of *Penicillium* are commonly present in *F. polyctena* living environment and possibly co−occur with ants inside their nests. It is, however, quite surprising that representatives of *Aspergillus*—a genus that similarly to *Penicillium* is known to efficiently degrade plant material [87] and also belongs to the Aspergillaceae family—were not found in this study at all.

*Penicillium* spp. are also well known for their antibiotic properties [88], and thus can play a special role in the interactions with ants as protective agents against potentially harmful microorganisms. It has been proven that the growth of *Aspergillus* spp. can be inhibited by the presence of *Penicillium* colonies [89]; therefore, the frequent occurrence of *Penicillium* on ant bodies, observed in this study, may additionally explain the absence of *Aspergillus* spp. among the fungal isolates of the same origin. Even more interestingly, other studies have shown that *Aspergillus* species can be pathogenic toward insects [90].

*Trichoderma* was the second most abundant fungal genus among the isolates from *F. polyctena* ants. Representatives of this taxon are known to be saprotrophs, decomposing plant and fungal matter [91,92], including coniferous needles, as indicated by their common occurrence in coniferous forest litter [93]. Considering that a red wood ants’ nest is an ecological isle of accumulation of plant material, especially including coniferous needles, the prevalence of *Trichoderma* on *F. polyctena* ants does not come as a surprise.

Among the yeasts isolated from ants’ bodies, we observed *Schwanniomyces* to be the most abundant genus. Interestingly, *Schwanniomyces* spp. have also been frequently isolated from other ant species: *F. rufa* [49], *F. aquilonia* [48] and *Camponotus vicinus* [21]. Moreover, Mankowski and Morrell showed that the addition of *Schwanniomyces polymorphus* to the basal diet of *C. vicinus* ants resulted in an increase in their weight [21]. *Schwanniomyces* representatives are known to produce B vitamins and break down various oligosaccharides into simple sugars, which indicates that they may be a good source of nutrients for ants [94,95]. It would be greatly beneficial for further studies to focus on this particular yeast genus in the context of ant–fungal interactions.

Another fungal taxon, isolated from *F. polyctena* in this study, as well as from the infrabuccal pellets of other ant species (*Formica rufa* and *Camponotus* spp.) in past studies, is *Entomortierella beljakovae* (previously *Mortierella beljakovae* [69]) [20]. A known distinctive trait of this species is the ability to produce inflated cells filled with prominent oil droplets, called gemmae [96]. We believe that those structures could be preferred by ants as supplemental food. Especially because our preliminary observations suggest that *F. polyctena* ants perform feeding behavior towards hyphae of *E. beljakovae* when confronted with them in the wild [97]. The relatively frequent presence of *Mucor* spp. spores on ants could be the result of ants foraging on organic−rich substrates (e.g., excrement), but also of the presence of these fungi inside nests.

Apart from *Aspergillus,* it was rather unexpected to find many fungal taxa common in coniferous forest litter, such as *Alternaria*, *Cladosporium* or *Fusarium,* to be very rare among our isolates from *F. polyctena* ants. A likely explanation for this would be that the *F. polyctena* nest material, filled with resin and sprayed with formic acid, may be an unsuitable habitat for certain fungal genera, including those mentioned above. This observation also further suggests that the mycobiota of the actively shaped *F. polyctena* ants’ environment, similarly to the mycobiota of *F. exsecta* nests [24], differ from the mycobiota of its surroundings; however, further studies on this subject are necessary to fully understand this phenomenon. Finally, because of the methodology we applied, some slow−growing fungal taxa could have been quantitatively underestimated, or even completely missing from the record in our study. The next step of this investigation will involve high−throughput sequencing of the ITS rDNA amplicons in order to circumvent the methodological constraints of a culturing−dependent study.

## 5. Conclusions

Red wood ants’ nests are specific, actively shaped micro−environments, which most likely host a mycobiota different from that of the surrounding forest litter and soil. Having analyzed a set of over 600 fungal strains isolated from the bodies of *Formica polyctena* ants, we conclude that the fungi from genera *Penicillium*, *Trichoderma*, *Mucor*, *Schwanniomyces* and *Entomortierella* commonly co−occur in those ants’ immediate surroundings. Considering that the diverse *Penicillium* spp. were the most common among all fungi we isolated from ants, we believe that certain species of this genus may be better adapted to survive in the ants’ nest environment than the other fungal taxa. Further studies on this subject will help us analyze the specificity and character of this insect–fungi interaction. Additionally, some fungal genera abundantly found in forest litter, such as *Aspergillus* and *Alternaria,* were found to be surprisingly rare among the isolates from *F. polyctena* ants. This, in turn, raises a question if the ant−made environment is not a suitable habitat for these fungal taxa, or if they are eliminated, either actively by insects, or indirectly, by means of competitive exclusion by other fungi, engaged in symbiosis with ants.

## Data Availability

The sequences obtained in this study were deposited in the NCBI GenBank database. Voucher specimens were deposited in the Herbarium of the Faculty of Biology, University of Warsaw. GenBank accession numbers and Herbarium collection numbers are available in the Supplementary Materials Table S2. More data are available upon request from the first author.

## Supplementary Materials

The following are available online at https://www.mdpi.com/2075-4450/12/3/204/s1, Table S1: Coordinates of sample sites (*Formica polyctena* anthills, from which ants were collected), Table S2: List of fungi isolated from *Formica polyctena* ants’ cadavers.
