# Supplementary material for: Chance or Necessity—The Fungi Co−Occurring with Formica polyctena Ants
Source: Insects. 2021 Feb 28;12(3):204. doi: 10.3390/insects12030204 (PMC7997191; doi:10.3390/insects12030204)
Supplement: Supplementary file 1 [file insects-12-00204-s001.pdf]

Table S1. Coordinates of sample sites (*Formica polyctena* anthills, from which ants were collected).

Mound ID:	Latitude:	Longitude:	Forest type:
113	54°48'6.51"N	17°53'59.46"E	coniferous
114	54°48'7.15"N	17°54'0.99"E	coniferous
115	54°46'43.79"N	17°51'53.22"E	coniferous
116	54°46'50.47"N	17°51'52.22"E	coniferous
117	54°46'50.32"N	17°51'55.80"E	coniferous
118	54°48'32.17"N	17°50'15.71"E	coniferous
119	54°48'33.35"N	17°50'16.84"E	coniferous
121	54°46'7.16"N	17°48'22.25"E	mixed
123	54°46'16.65"N	17°57'33.22"E	mixed
125	54°46'14.98"N	17°57'45.10"E	mixed
126	54°45'22.47"N	18° 0'31.01"E	mixed
127	52°15'43.78"N	20°53'31.05"E	mixed
128	52°15'44.54"N	20°53'29.04"E	mixed
130	52°15'46.06"N	20°53'23.70"E	mixed
131	52°15'46.24"N	20°53'24.34"E	mixed
132	52°15'46.48"N	20°53'22.57"E	mixed
133	52°15'46.76"N	20°53'21.66"E	mixed
134	52°15'47.47"N	20°53'19.48"E	mixed

Table S2. List of fungi isolated from *Formica polyctena* ants' cadavers.

Subphylum	Isolated taxa	Herbarium ID number	Morphotype number	GenBank Accession Number
Agaricomycotina	*Apiotrichum sp.*	WA50774	F8	MW577193
Mortierellomycotina	*Entomortierella beljakovae*	-	F10	MW550308
Mortierellomycotina	*Entomortierella sp.*	-	F59	MW553074
Mucoromycotina	*Absidia sp.*	WA50772	F11	MW577195
Mucoromycotina	*Mucor abundans*	WA50749	F44	MW577216
Mucoromycotina	*Mucor plumbeus*	WA50765	F18	-
Mucoromycotina	*Mucor sp.* ('Mucor racemosus group')	WA50735	F76	-
Mucoromycotina	*Mucor sp.* ('Mucor racemosus group')	WA50751	F40	MW577214
Mucoromycotina	*Rhizopus sp.*	-	F49	-
Mucoromycotina	*Rhizopus sp.*	-	F81	-
Mucoromycotina	*Rhizopus sp.*	-	F46	MW577349
Mucoromycotina	*Umbelopsis sp.*	WA50764	F20	MW577201
Pezizomycotina	*Akanthomyces sp.*	WA50741	F62	MW577223
Pezizomycotina	*Alternaria sp.*	WA50744	F54	MW577220
Pezizomycotina	*Arthrinium sp.*	WA50743	F56	MW577221
Pezizomycotina	*Botrytis sp.*	WA50740	F65	MW577224
Pezizomycotina	*Chaetomiaceae sp.*	WA50725	F113	MW577237
Pezizomycotina	*Arcopilus sp.*	WA50771	F5	MW577191

Pezizomycotina	*Didymellaceae sp.*	WA50723	F117	MW577238
Pezizomycotina	*Dothioraceae sp.*	WA50722	F118	MW577239
Pezizomycotina	*Fusarium sp.*	-	F7	MW550307
Pezizomycotina	*Fusarium sp.*	WA50770	F13	MW577196
Pezizomycotina	*Penicillium sp.*	WA50730	F91	MW577233
Pezizomycotina	*Penicillium soppii*	WA50779	F1	MW577188
Pezizomycotina	*Penicillium sp.*	WA50724	F114	MW575451
Pezizomycotina	*Penicillium sp.*	WA50737	F69	MW577227
Pezizomycotina	*Penicillium sp.*	WA50742	F61	MW577222
Pezizomycotina	*Penicillium sp.*	WA50748	F45	MW577217
Pezizomycotina	*Penicillium sp.*	WA50753	F38	MW577212
Pezizomycotina	*Penicillium sp.*	WA50757	F31	MW577208
Pezizomycotina	*Penicillium sp.*	WA50767	F16	MW577199
Pezizomycotina	*Penicillium sp.* (sct. Aspergilloides)	-	F28	MW550309
Pezizomycotina	*Penicillium sp.* (sct. Aspergilloides)	-	F29	MW550310
Pezizomycotina	*Penicillium sp.* (sct. Aspergilloides)	-	F98	MW550316
Pezizomycotina	*Penicillium sp.* (sct. Aspergilloides)	WA50734	F78	MW577229
Pezizomycotina	*Penicillium sp.* (sct. Aspergilloides)	WA50752	F39	MW577213
Pezizomycotina	*Penicillium sp.* (sct. Aspergilloides)	WA50755	F36	MW577210
Pezizomycotina	*Penicillium sp.* (sct. Aspergilloides)	WA50761	F23	MW577204
Pezizomycotina	*Penicillium sp.* (sct. Aspergilloides)	WA50762	F22	MW577203
Pezizomycotina	*Penicillium sp.* (sct. Aspergilloides)	WA50777	F3	MW577189
Pezizomycotina	*Penicillium sp.* (sct. Brevicompacta)	WA50732	F87	MW577231
Pezizomycotina	*Penicillium sp.* (sct. Brevicompacta)	WA50736	F71	MW577228
Pezizomycotina	*Penicillium sp.* (sct. Brevicompacta)	WA50750	F43	MW577215
Pezizomycotina	Penicillium sp. (sct. Brevicompacta)	WA50773	F9	MW577194
Pezizomycotina	*Penicillium sp.* (sct. Citrina)	WA50760	F25	MW577205
Pezizomycotina	*Penicillium sp.* (sct. Exilicaulis)	WA50733	F84	MW577230
Pezizomycotina	*Penicillium sp.* (sct. Exilicaulis)	WA50776	F4	MW577190
Pezizomycotina	*Penicillium sp.* (sct. Fasciculata)	WA50739	F66	MW577225
Pezizomycotina	*Penicillium sp.* (sct. Fasciculata)	WA50766	F17	MW577200
Pezizomycotina	*Penicillium sp.* (sct. Lanata-Divaricata)	WA50763	F21	MW577202
Pezizomycotina	*Pseudogymnoascus sp.*	WA50729	F94	MW577234
Pezizomycotina	*Scorias sp.*	-	F107	MW550318
Pezizomycotina	*Talaromyces sp.*	WA50759	F26	MW577206
Pezizomycotina	*Trichoderma sp.* (sct. Trichoderma)	-	F42	MW550311
Pezizomycotina	*Trichoderma sp.* (sct. Trichoderma)	-	F48	MW550312
Pezizomycotina	*Trichoderma sp.* (sct. Trichoderma)	-	F53	MW550313
Pezizomycotina	*Trichoderma sp.* (sct. Trichoderma)	-	F74	MW550314
Pezizomycotina	*Trichoderma sp.* (sct. Trichoderma)	-	F92	MW550315
Pezizomycotina	*Trichoderma sp.* (sct. Trichoderma)	-	F104	MW550317
Pezizomycotina	*Trichoderma sp.* (sct. Trichoderma)	WA50726	F109	MW577236
Pezizomycotina	*Trichoderma sp.* (sct. Trichoderma)	WA50731	F90	MW577232
Pezizomycotina	*Trichoderma sp.* (sct. Trichoderma)	WA50745	F52	MW577219

Pezizomycotina	*Trichoderma sp.* (sct. Trichoderma)	WA50754	F37	MW577211
Pezizomycotina	*Trichoderma sp.* (sct. Trichoderma)	WA50756	F32	MW577209
Pezizomycotina	*Trichoderma sp.* (sct. Trichoderma)	WA50768	F15	MW577198
Pezizomycotina	*Trichoderma sp.* (sct. Trichoderma)	WA50769	F14	MW577197
Pezizomycotina	*Trichoderma sp.* (sct. Trichoderma)	WA50775	F6	MW577192
Saccharomycotina	*Candida sp.*	WA50721	F120	MW553041
Saccharomycotina	*Candida sp.*	WA50727	F103	MW575450
Saccharomycotina	*Schwanniomyces sp.*	WA50728	F95	MW577235
Saccharomycotina	*Schwanniomyces sp.*	WA50738	F68	MW577226
Saccharomycotina	*Schwanniomyces sp.*	WA50746	F51	MW577218
Saccharomycotina	*Schwanniomyces sp.*	WA50747	F50	MW592383
Saccharomycotina	*Schwanniomyces sp.*	WA50758	F30	MW577207
